# Exogenous Delivery of Link N mRNA into Chondrocytes and MSCs—The Potential Role in Increasing Anabolic Response

**DOI:** 10.3390/ijms20071716

**Published:** 2019-04-06

**Authors:** Gauri Tendulkar, Sabrina Ehnert, Vrinda Sreekumar, Tao Chen, Hans-Peter Kaps, Sonia Golombek, Hans-Peter Wendel, Andreas K. Nüssler, Meltem Avci-Adali

**Affiliations:** 1 Siegfried Weller Institute for Trauma Research at the BG Trauma Center, Eberhard Karls Universität Tübingen, Schnarrenbergstraße 95, 72076 Tübingen, Germany; sabrina.ehnert@gmail.com (S.E.); vrindaskumar@gmail.com (V.S.); zzuchentao@yahoo.com (T.C.); hpkaps@gmx.de (H.-P.K.); andreas.nuessler@gmail.com (A.K.N.); 2 Department of Thoracic and Cardiovascular Surgery, University Hospital Tübingen, Calwerstraße 7/1, 72076 Tübingen, Germany; sonia.golombek@klinikum.uni-tuebingen.de (S.G.); hans-peter.wendel@med.uni-tuebingen.de (H.-P.W.); meltem.avci-adali@klinikum.uni-tuebingen.de (M.A.-A.)

**Keywords:** Synthetic mRNA, Link N peptide, mRNA delivery, musculoskeletal disorders

## Abstract

Musculoskeletal disorders, such as osteoarthritis and intervertebral disc degeneration are causes of morbidity, which concomitantly burdens the health and social care systems worldwide, with massive costs. Link N peptide has recently been described as a novel anabolic stimulator for intervertebral disc repair. In this study, we analyzed the influence on anabolic response, by delivering synthetic Link N encoding mRNA into primary human chondrocytes and mesenchymal stromal cells (SCP1 cells). Furthermore, both cell types were seeded on knitted titanium scaffolds, and the influence of Link N peptide mRNA for possible tissue engineering applications was investigated. Synthetic modified Link N mRNA was efficiently delivered into both cell types and cell transfection resulted in an enhanced expression of *aggrecan*, *Sox 9*, and *type II collagen* with a decreased expression of *type X collagen*. Interestingly, despite increased expression of *BMP2* and *BMP7*, BMP signaling was repressed and TGFβ signaling was boosted by Link N transfection in mesenchymal stromal cells, suggesting possible regulatory mechanisms. Thus, the exogenous delivery of Link N peptide mRNA into cells augmented an anabolic response and thereby increased extracellular matrix synthesis. Considering these findings, we suppose that the cultivation of cells on knitted titanium scaffolds and the exogenous delivery of Link N peptide mRNA into cells could mechanically support the stability of tissue-engineered constructs and improve the synthesis of extracellular matrix by seeded cells. This method can provide a potent strategy for articular cartilage and intervertebral disc regeneration.

## 1. Introduction

Osteoarthritis (OA) and intervertebral disc (IVD) degeneration are primarily caused by an imbalance between the synthesis and degradation of extracellular matrix (ECM) [1]. They belong to musculoskeletal disorders with the world’s largest socioeconomic impact, which are certainly accompanied by joint pain and lower-back pain (LBP) [2], consequently affecting the quality of life [3]. Musculoskeletal debility, especially LBP is the most common health problem seen in the elderly population worldwide, with a global prevalence of 65% [4], and the highest contribution to physical disability [5]. Therefore, non-pharmacological or pharmacological and non-invasive or invasive therapies [6] have been widely used to reduce the degree of pain. Despite progress, the treatment of OA and IVD degeneration remains a challenge [6], due to lack of structural and biomechanical restoration [7,8,9]. Thus, alternative treatments are required to restore the functionality of damaged tissue. Although, there are substantial differences regarding the anatomy and embryonic origin between human IVDs and articular joints, there are great similarities in terms of matrix-degrading factors and the response to mechanical loading in both types of joints [10].

In recent years, gene therapy has been widely explored in tissue engineering to produce specific growth factors, transcription factors, or molecules with therapeutic effects [9,11] to stimulate or induce regenerative responses in the tissue. Although viral- and plasmid-mediated gene delivery has demonstrated the ability to effectively produce desired proteins, it also has several disadvantages, such as high risk of insertional mutagenesis leading to tumorigenicity due to genome integration, high immunogenicity, inefficient transduction in non-dividing cells, high levels of pre-existing immunity, and other potential serious complications [12,13]. Therefore, in recent years, the synthetic messenger RNA (mRNA)-based protein expression in cells is gaining much attention. The use of synthetic mRNA abrogates the risk of integration to the host genome [13]. The translational efficacy of synthetic mRNA has also improved compared to plasmids, since it bypasses the need for nuclear trafficking and immediately translated upon entering the cytoplasm. In addition, chemical modification of desired mRNAs has elevated stability and significantly reduced immunogenicity [13]. In a previous study, Aini et al. demonstrated the delivery of mRNA encoding the cartilage anabolic transcription factor runt-related transcription factor 1 (RUNX1) as a promising osteoarthritis treatment option [14]. The injection of RUNX1 mRNA into mouse OA-knee joints significantly suppressed the progression of OA compared to the non-treated control. Moreover, it showed augmented expression of cartilage-anabolic markers and proliferation in articular chondrocytes, initiating a transcriptional cascade for therapeutic effects within chondrocytes.

Link protein is a membrane bound and constitutively expressed glycoprotein that stabilizes the interaction between aggrecan and hyaluronan [15] in extracellular matrices including IVD. The N terminal proteolytic cleavage of Link protein generates a Link N peptide, which is a 16 amino acid long peptide [15]. In previous studies, the Link N peptide has been identified as a promising growth factor that stimulates ECM synthesis in IVDs [16,17,18]. The Link N peptide appears to have an effect on IVD metabolism, which stimulates the proteoglycan synthesis [18]. Furthermore, Antoniou et al. and Mwale et al. demonstrated that Link N has suitable properties to drive chondrogenesis, and therefore, serves as a potential growth factor during IVD healing [17,19,20,21].

In this study, we examined the influence of the exogenous delivery of synthetic mRNA encoding Link N peptide on the anabolic response in human primary chondrocytes and bone marrow-derived mesenchymal stromal cells (SCP1 cells). Therefore, synthetic mRNA encoding Link N was generated and the transfection efficiency was analyzed in both cell types. Functional Link N mRNA delivery augmented the anabolic response in primary human chondrocytes and SCP1 cells, promoting extracellular matrix related gene expression in 2D cell cultures as well as in 3D cultures after seeding cells on knitted titanium scaffolds [22]. Thus, the delivery of synthetic Link N mRNA into chondrocytes and MSCs presents a new therapeutic strategy for augmenting the anabolic response of cells for improving cartilage and/or IVDs regeneration.

## 2. Results

### 2.1. Generation of Synthetic Modified Link N mRNA

**Figure 1:**
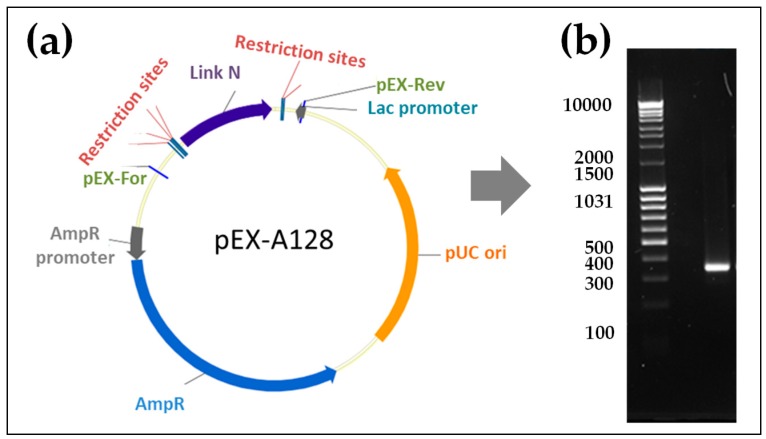
Synthesis of modified Link N mRNA. (**a**) Plasmid insert encoding the Link N peptide was amplified using PCR, and a poly T120 tail was added. The DNA product was then transcribed in vitro into mRNA. (**b**) The generated mRNA product was purified and the specific length and purity were confirmed by agarose gel (1%) electrophoresis. Right Lane: Link N mRNA (351 nucleotides) after the purification, left Lane: 0.5–10 kB RNA ladder.

To generate the Link N mRNA, we performed a PCR with the template pEX-A128 vector containing a Link N encoding sequence, this allowed us to amplify and add a poly T-tail to the Link N encoding sequence. DNA was then transcribed in vitro into mRNA, and mRNA purity was demonstrated using 1% agarose gel electrophoresis. The purified Link N mRNA showed the expected length of 351 nucleotides (Figure 1).

### 2.2. Analysis of Transfectability of Human Primary Chondrocytes and SCP1 Cells with Synthetic mRNA

**Figure 2:**
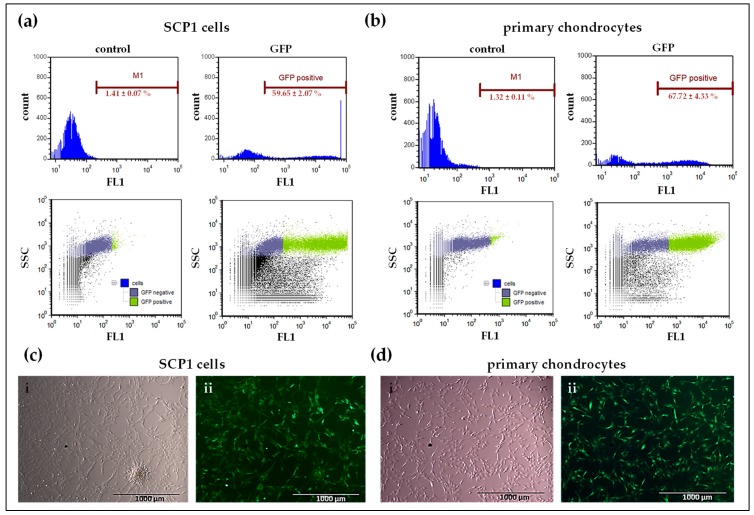
Analysis of transfection efficiency using eGFP mRNA in SCP1 cells and human primary chondrocytes. Flow cytometry analyses of (**a**) SCP1 cells and (**b**) human primary chondrocytes, 24 h post transfection with 1 µg eGFP mRNA using Lipofectamine 2000. Cells treated with Lipofectamine 2000 were used as controls. Representative fluorescence microscopic images of eGFP mRNA transfected (**c**) SCP1 cells and (**d**) human primary chondrocytes: (i) phase contrast bright field image; (ii) GFP channel image of eGFP mRNA transfected cells. Scale bar = 100 µm.

To confirm the transfectability of human primary chondrocytes as well as SCP1 cells using synthetic mRNA, first, in vitro transcribed (IVT) mRNA encoding eGFP was used. Transfection efficiency was analyzed using flow cytometry (Figure 2a,b) and the production of eGFP was confirmed using epifluorescence microscopy (Figure 2c,d). Flow cytometry revealed that 24 h after eGFP mRNA transfection, 59.65 ± 2.07% of SCP1 cells and 67.72 ± 4.33% of primary chondrocytes expressed eGFP. The successful mRNA transfection was easily investigated in primary human chondrocytes as well as in SCP1 cells using eGFP mRNA. Compared to the control (cells treated with Lipofectamine 2000), the transfection of 100,000 cells with 1 µg mRNA, complexed with Lipofectamine 2000, was sufficient to induce a significant increase in protein expression.

**Figure 3:**
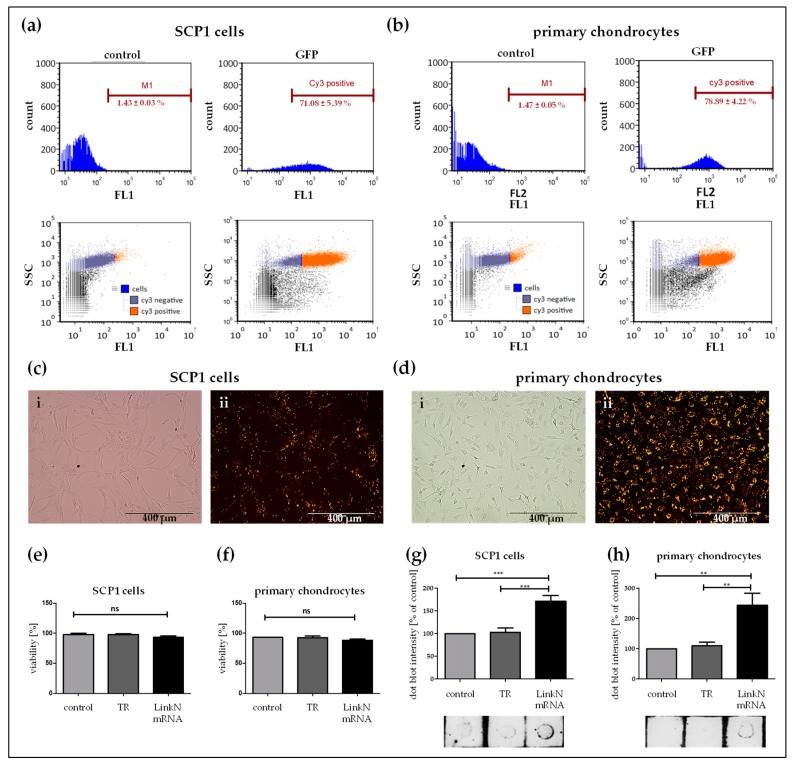
Analysis of Link N mRNA transfection efficiency in SCP1 cells and primary chondrocytes, and the influence of Link N mRNA transfection on cell viability. (**a**,**c**,**e**,**g**) SCP1 cells and (**b**,**d**,**f**,**h**) human primary chondrocytes were analyzed 24 h post-transfection with 1 µg Cy3 labeled Link N mRNA using flow cytometry. Representative fluorescence microscopic images of Link N mRNA transfected cells. (i) Phase contrast bright field image, (ii) Cy3 channel image of Cy3 labeled Link N mRNA. Scale bar = 400 µm. In vitro, cells transfected with 1 µg of unlabeled Link N mRNA did not exhibit any adverse viability effects. Using NucleoCounter (NC-200), cell viability was assessed 24 h post-transfection. Acridine Orange and DAPI double staining identified the percentage of viable cells. Dot blot quantification of the link N peptide in the supernatant fraction of cells treated with Lipofectamine 2000, eGFP mRNA, and Link N mRNA in the cells. Untransfected cells, and cells transfected only with the transfection reagent (TR) Lipofectamine 2000, were used as controls. Data are represented as mean + SEM (N ≥ 3) and were compared using one-way ANOVA with Bonferroni’s multiple comparison test. *** *p* < 0.001, ** *p* < 0.01, ns: not significant.

Similarly, the Link N mRNA transfection efficiency was analyzed in SCP1 cells and human primary chondrocytes using flow cytometry (Figure 3a,b) and fluorescence microscopy (Figure 3c,d). Therefore, 1 µg of Cy3-labelled Link N mRNA was used to generate the transfection complexes and Cy3 labeled Link N mRNA was successfully delivered into 71.08 ± 5.39% of SCP1 cells and 78.89 ± 4.22% of primary chondrocytes.

The produced Link N peptide was detected using the dot blot assay (Figure 3g,f). The superior amount of Link N peptide observed seems to correspond to the effect of transfection. Increased dot intensity was seen 24h post Link N mRNA transfection when compare to eGFP mRNA transfection and cells treated with Lipofectamine 2000. As seen with the dot blot intensity quantification, no notable differences were observed between the SCP1 cells or primary chondrocytes treated with Lipofectamine 2000 and eGFP mRNA.

### 2.3. Exogenous Delivery of Link N mRNA Does Not Affect Cell Viability

To verify that Link N mRNA delivery into cells is not detrimental, cell viability was assessed. Human primary chondrocytes and SCP1 cells were transfected with 1 µg of Link N mRNA, and 24 h post-transfection, double staining using Acridine Orange and DAPI determined the percentage of viable cells. For both cell types, a cell viability of >96% was detected (Figure 3e,f), and cell viability was not significantly different from the control cells.

### 2.4. Link N mRNA Transfection Augments Anabolic Effects in Human Primary Chondrocytes

**Figure 4:**
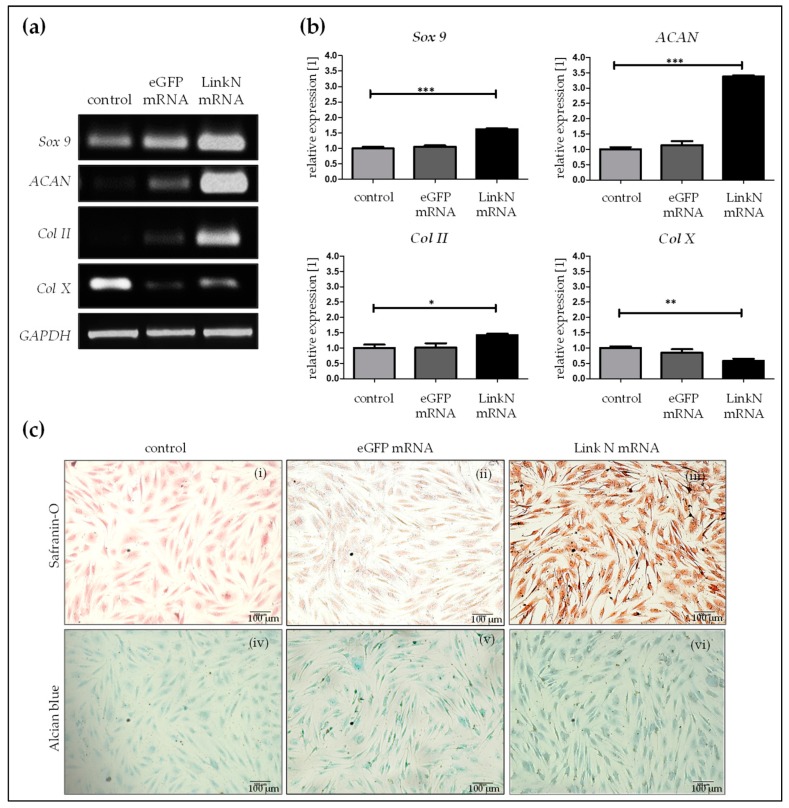
Influence of Link N mRNA delivery into primary chondrocytes on aggrecan (*ACAN*), *Sox 9*, collagen type II (*Col II*) and type X (*Col X*) gene expression, and production of collagen and proteoglycan matrix. Primary human chondrocytes (1 × 10^5^) were transfected with 1 µg Link N mRNA, and the influence on the expression of chondrocyte anabolic markers was investigated. (**a**) Representative gel images evaluating changes in *ACAN*, *Sox 9*, *Col II*, and *Col X* gene expression, 24 h after Link N mRNA transfection. (**b**) Gene expression examined 24 h after Link N mRNA transfection, using semi-quantitative RT-PCR. Cells treated with Lipofectamine 2000 and eGFP mRNA transfected cells were used as controls. *GAPDH* was used as an internal control and served to normalize the expression. Densitometric analyses are represented as mean ± SEM (N≥3) and were compared using one-way ANOVA with Bonferroni’s multiple comparison test (*** *p*< 0.001, ** *p*< 0.01, * *p* < 0.05). (**c**) The expression of collagen and proteoglycan matrix proteins was examined in cells treated with Lipofectamine 2000 alone (i,iv), eGFP mRNA (ii,v), and Link N mRNA transfected (iii,vi) human primary chondrocytes using safranin O and alcian blue-staining 7 days post-transfection. In comparison to the control, Link N mRNA transfected cells showed the presence of an intensely stained matrix. Scale bar = 100 µm.

Influence of the delivered Link N mRNA on chondrocyte anabolic markers was investigated by performing semi-quantitative reverse transcription-polymerase chain reaction (RT-PCR) 24 h post-transfection of human primary chondrocytes with 1 µg Link N mRNA. Densitometric analyses revealed that, compared to cells treated with Lipofectamine 2000 and cells transfected with 1 µg eGFP mRNA, the transfection of cells with 1 μg Link N mRNA resulted in a significant increase in chondrocyte-specific markers, aggrecan (*ACAN*), *Sox 9*, and *Col II* (Figure 4a). The expression of *Sox 9* (*** *p* < 0.001), a master transcription factor for the specification and maintenance of cartilage, and *ACAN* (*** *p* < 0.001), which is an ECM specific marker for cartilage, was significantly increased up to 1.6 and 3.4-fold, respectively, upon Link N mRNA transfection compared with the cells treated with Lipofectamine 2000 or cells transfected with eGFP mRNA. Similarly, gene expression of *type II collagen* (* *p* < 0.05), a major cartilage ECM protein, also increased by 1.4-fold in the Link N mRNA transfected group. However, gene expression of *Col X* (** *p* < 0.01), a hypertrophic marker, was significantly downregulated to 0.6-fold in the Link N mRNA-transfected group compared to cells treated with Lipofectamine 2000 or cells transfected with eGFP mRNA.

Furthermore, the accumulation of chondrocyte-specific ECM upon Link N mRNA delivery was examined 7 days post-transfection. Alcian blue and safraninO staining were used to investigate the expression of proteoglycans and collagen, respectively. Safranin O staining showed an intense red color in Link N mRNA transfected cells compared to cells treated with Lipofectamine 2000 and/or eGFP mRNA. The formation of GAGs was confirmed using Alcian blue dye, which resulted in an intense blue color in Link N mRNA transfected cells compared to cells treated with Lipofectamine 2000—control cells (Figure 4b) and also with eGFP mRNA. These results indicate a positive effect of Link N mRNA transfection on ECM metabolism of chondrocytes, and show agreement with the results of gene expression analyses.

### 2.5. Link N mRNA Transfection Upholds the Expression of ECM-Related Genes in SCP1 Cells

**Figure 5:**
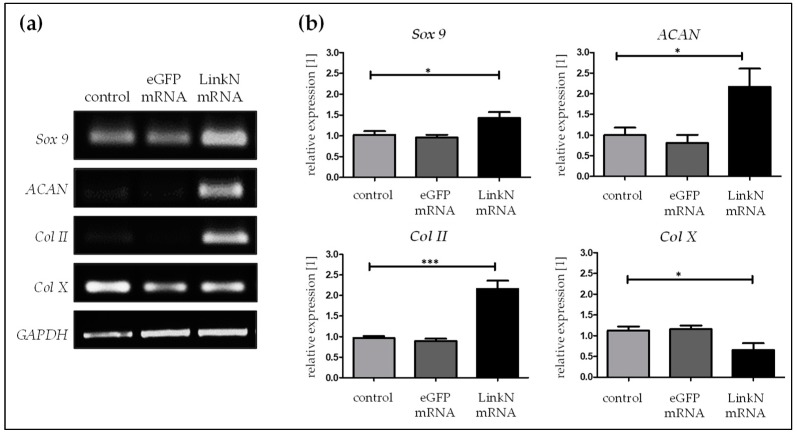
Influence of Link N mRNA delivery into SCP1 cells on aggrecan (*ACAN*), *Sox 9*, type II and type X collagen gene expression.SCP1 cells (1 × 10^5^) were transfected with 1 µg of Link N mRNA, and the influence on the expression of chondrocyte-specific markers was investigated. (**a**) Representative gel images evaluating the changes in *ACAN*, *Sox 9*, *Col II* and *Col X* gene expression, 24 h after Link N mRNA transfection. (**b**) Changes in *ACAN*, *Sox 9*, *Col II* and *Col X* gene expression were examined 24 h after Link N mRNA transfection, using semi-quantitative RT-PCR. Cells treated with Lipofectamine 2000 and eGFP mRNA transfected cells were used as controls. *GAPDH* was used as an internal control and served to normalize the expression. Relative expression levels obtained by densitometric analyses are represented as mean ± SEM (N = 3) and were compared using one-way ANOVA with Bonferroni’s multiple comparison test (*** *p* < 0.001, * *p* < 0.05).

To investigate the response of MSCs to the synthetic Link N mRNA treatment, SCP1 cells were transfected with 1 µg of Link N mRNA, and semi-quantitative RT-PCR was performed 24 h post transfection. A significantly higher expression of the chondrocyte-specific markers *Sox 9* (* *p* < 0.05), *ACAN* (* *p* < 0.05), and *Col II* (*** *p* < 0.001) was observed in Link N mRNA transfected cells compared to cells treated with Lipofectamine 2000 or cells transfected with eGFP mRNA (Figure 5).

Although, synthetic Link N mRNA delivery in SCP1 cells increased *Sox 9* expression to 1.3-fold, a stronger effect was seen on *collagen II* and *aggrecan* with 2.2-fold change increase for both compared to cells treated with Lipofectamine 2000 or cells transfected with eGFP mRNA. In addition, the *Col X* (* *p* < 0.05) gene expression was significantly down-regulated to 0.6-fold upon Link N mRNA transfection. Overall, synthetic Link N mRNA delivery into SCP1 cells upholds the expression of ECM related genes.

**Figure 6:**
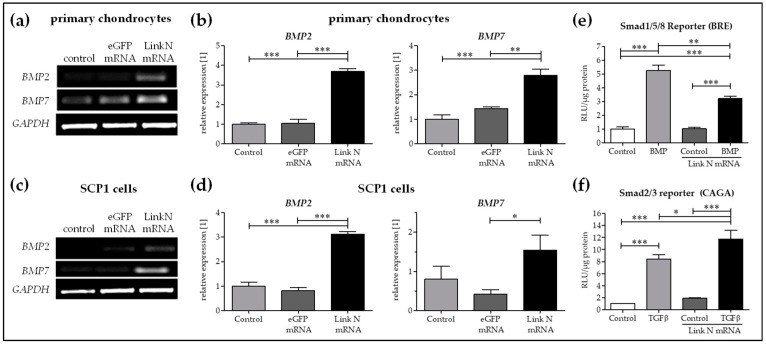
The influence of Link N mRNA delivery into primary chondrocytes and SCP1 cells on *BMP2* and *BMP7* gene expression. Primary chondrocytes and SCP1 cells (1 × 10^5^) were transfected with 1 µg of Link N mRNA, and the influence on *BMP2* and *BMP7* expression was examined 24 h after Link N mRNA transfection, using semi-quantitative RT-PCR. Cells treated with Lipofectamine 2000 and eGFP mRNA transfected cells were used as controls. *GAPDH* was used as an internal control and served to normalize the expression. (**a**,**c**) Representative gel images to evaluate the changes in *BMP2* and *BMP7* gene expression, 24 h after Link N mRNA transfection. (**b**,**d**) Relative expression levels obtained by densitometric analysis. Link N mRNA transfected SCP1 cells infected with (**e**) Ad5-BRE-Luc reporter construct (smad1/5/8 reporter) and stimulated with 50 ng/mL rhBMP 2 or with (**f**) Ad5-CAGA9-MLP-Luc reporter construct (smad2/3 reporter) and stimulated with 10 ng/mL rhTGFβ1. After 24 h, luciferase activity was measured in cell lysates and normalized to total protein content. Data are represented as mean ± SEM (N = 3) and were compared using one-way ANOVA with Bonferroni’s multiple comparison test. *** *p* < 0.001, ** *p* < 0.01, * *p* < 0.05 as indicated.

Promotion and maintenance of aggrecan and collagen II appears to be mainly regulated by bone morphogenetic proteins (BMPs) including BMP2 and BMP7 in SCP1 cells and human primary chondrocytes. A significantly higher expression of *BMP2* and *BMP7* was observed in Link N transfected cells compared to cells transfected with eGFP mRNA or treated with Lipofectamine 2000 (Figure 6a–d). Furthermore, to investigate the induction of differentiation upon Link N mRNA transfection in SCP-1 cells, hypothesizing through BMP signaling, a reporter assay was performed. Therefore, Link N mRNA transfected SCP1 cells were infected with Ad5-BRE-Luc adenoviral particles (Smad1/5/8 reporter construct) and stimulated for 24 h with 50 ng/mL of recombinant human BMP2. Luciferase activity was measured in cell lysates. Link N mRNA transfection alone didnot induce Smad1/5/8 signaling in mesenchymal stromal cells (Figure 6e). Link N mRNA transfected SCP1 cells were then infected with Ad5-CAGA_9_-MLP-Lucadenoviral particles (Smad2/3 reporter construct) and stimulated for 24 h with 10 ng/ml of recombinant human TGFβ. Luciferase activity was measured in cell lysates. Adding rhTGFβ_1_ jointly stimulated Smad2/3 signaling (Figure 6f) and hence we speculate that despite the increase in BMP expression, TGFβ inhibited the BMP response in mesenchymal stromal cells.

### 2.6. Transfectability of Cells Seeded on Knitted Titanium Scaffold with eGFP mRNA

**Figure 7:**
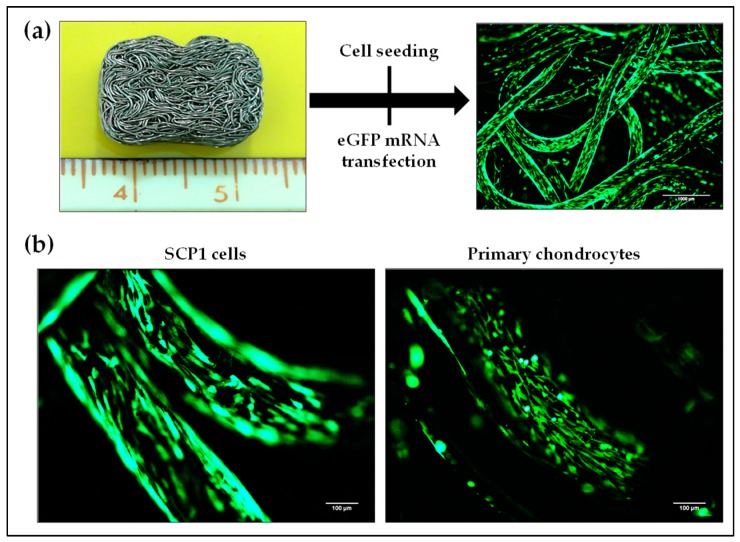
eGFP mRNA transfection of cells seeded on knitted titanium scaffolds. (**a**) Illustration of cell seeding on the knitted titanium scaffolds, and the following eGFP mRNA transfection. (**b**) Positive eGFP expression of SCP1 cells and human primary chondrocytes adhered to knitted titanium scaffold confirmed successful mRNA transfection. Scale bar = 100 µm.

Biocompatibility is a fundamental prerequisite for any biomaterial to be used in tissue engineering. Thus, a knitted titanium scaffold, which showed in our previous studies a high biocompatibility, was used as an example for the 3D cultivation of cells for potential application in IVD treatment [23]. SCP1 cells and primary human chondrocytes were seeded on these biocompatible knitted titanium scaffolds and the transfectability of these cells was analyzed using lipoplexes-containing Lipofectamine 2000 and 1 µg eGFP mRNA. Figure 7a illustrates the over-expression of eGFP in cells (either SCP1 cells or human primary chondrocytes) seeded on the knitted titanium scaffold 24 h post-transfection. The successful eGFP mRNA transfection of SCP1 cells as well as human primary chondrocytes was demonstrated by detecting eGFP protein expression using fluorescence microscopy (Figure 7b).

### 2.7. Link N mRNA Transfection of Cells Seeded on Knitted Titanium Scaffold Triggers the Expression of Chondrocyte-Specific and ECM-Related Genes

**Figure 8:**
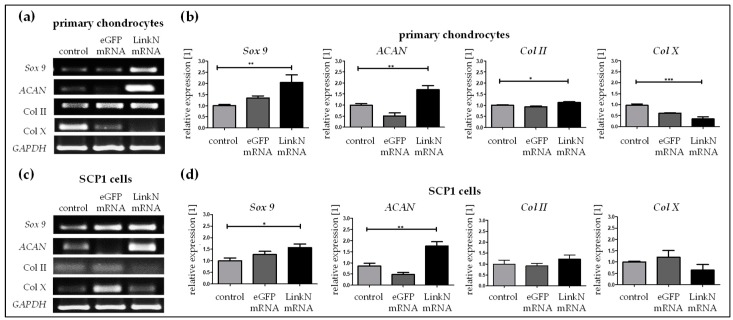
The effect of Link N mRNA transfection on cells seeded on knitted titanium scaffold- changes in *ACAN*, *Sox 9*, *Col II* and *Col X* gene expression. Primary human chondrocytes or SCP1 cells (1 × 10^5^) were transfected with 1 µg of Link N mRNA, and the influence on ECM-related genes and chondrocyte anabolic markers was investigated. Representative gel images evaluating the changes in *ACAN*, *Sox 9*, *Col II* and *Col X* gene expression 24 h after Link N mRNA transfection in (**a**) human primary chondrocytes and (**c**) SCP1 cells. Gene expression were examined 24 h after Link N mRNA transfection in (**b**) human primary chondrocytes and (**d**) SCP1 cells seeded on knitted titanium scaffolds using semi-quantitative RT-PCR. Cells treated with Lipofectamine 2000and eGFP mRNA transfected cells were used as controls. *GAPDH* was used as an internal control and served to normalize the expression. Relative expression levels obtained by densitometric analyses are represented as mean ± SEM (N ≥ 3), and were compared using one-way ANOVA with Bonferroni’s multiple comparison test (*** *p* < 0.001, ** *p* < 0.01, * *p* < 0.05).

Transfection with 1 µg of Link N mRNA was performed in SCP1 cells and human primary chondrocytes seeded on knitted titanium scaffolds. Functional potency was evaluated by analyzing the expression of aggrecan, *Sox 9*, and type II and type X collagen using semi-quantitative RT-PCR 24 h post Link N mRNA transfection. A 2-fold increase in *Sox 9* (** *p* < 0.01), 1.7-fold increase in *ACAN* (** *p* < 0.01), and 1.2-fold increase in *Col II* (* *p* < 0.05) gene expression was detected, confirming that Link N mRNA transfection can augment the anabolic effect in chondrocytes in 3D cultures (Figure 8a). Moreover, *Col X* (*** *p* < 0.001) gene expression was significantly down-regulated (0.3-fold).In SCP1 cells seeded on scaffolds, chondrocyte-specific genes *Sox 9* (* *p* < 0.05) and *ACAN* (** *p* < 0.01) were also significantly up-regulated to 1.6-fold and 1.8-fold, respectively, 24 h post transfection (Figure 8b), compared to cells treated with Lipofectamine 2000 or cells transfected with eGFP mRNA. The expression of collagen II tended to be up-regulated and collagen X was down-regulated after the Link N mRNA transfection, but these differences were not significant.

## 3. Discussion

In recent years, viral vector- and plasmid-based gene therapies have drawn much attention as alternatives to the administration of growth factors or peptides. However, the major problem remains the safety concerns [24,25]. Thus, the clinical application remains challenging [26]. In contrast, mRNA-based strategies have been shown to be a safer alternative for producing proteins of interest inside cells without integration into host genomes compared to viral- and plasmid-based delivery methods [13,27,28]. In addition, the stability of synthetic mRNA can be effectively increased, and the immune activation potential decreased by chemical modifications [29]. Generally, synthetic mRNA is degraded after 2–3 days of delivery [13]. In the present study, a functional synthetic mRNA encoding Link N was successfully generated to stimulate ECM production. Using a cationic lipid, the chemically modified mRNA encoding Link N was efficiently delivered into chondrocytes and SCP1 cells without any adverse effects on cell viability. As a result, Link N mRNA transfection enhanced the expression of cartilage specific ECM components *ACAN* and *Col II* in human primary chondrocytes and SCP1 cells, and further reduced *Col X* synthesis. We, therefore, assume that the transient genome integration free over-expression of Link N in chondrocytes and MSCs using synthetic mRNA may improve joint repair.

While there are great similarities between the expression profile of collagen and aggrecan in articular cartilage and IVDs, their ratios are different, respectively [30]. Moreover, there is a substantial difference regarding their distinct morphology and/or extracellular matrix expression profiles between the articular cartilage from joints and IVD tissue [31]. The progressive loss of ECM components, *Col II* and *ACAN*, primarily due to the increase in catabolic pathophysiological events is the hallmark of articular cartilage and IVD degeneration [30,32]. Nonetheless, remarkable up-regulation of collagen type X has also been reported to occur in articular cartilage and IVDs during osteoarthritis and low back pain, respectively [33]. It ultimately leads to cartilage or IVD destruction and loss of function. Therefore, the repair or replacement of these tissues is required. From the vast knowledge of cartilage anabolic factors, one of the anabolic agents, e.g., RUNX1, is able to induce cartilage regeneration [14]. Although various strategies have been initiated to attempt cartilage repair [34], their clinical therapeutic efficacy remains a major challenge in chronic joint diseases. Recently, the N terminal peptide of Link protein (Link N: DHLSDNYTLDHDRAIH) generated due to proteolytic cleavage, has been identified as a potential growth factor that plays a pivotal role in stimulating anabolic effects during IVD repair [16]. In addition, it has been demonstrated that Link N synthetic peptide treatment restores proteoglycan content, a prerequisite for IVD repair [19]. Moreover, injecting synthetic Link N into discs of a rabbit model of IVD degeneration resulted in partial restoration of disc height [21]. However, challenges associated with peptide drugs, such as interplay between the pharmacokinetics upon administration, immunogenicity, and formulation conditions, in addition to the rapid clearance from the body and conformational flexibility, limits their clinical use [35,36]. Moreover, the concentration of peptide drugs and their local activity is often not sufficient to support the later stages of tissue repair due to low bioavailability. On the contrary, nucleic acid therapy provides the steady release of the desired protein from cells [13].

In this study, for the first time, we analyzed the potential of the exogenous delivery of synthetic Link N mRNA to restore ECM content as a potential musculoskeletal therapeutic application. Influences of synthetic Link N mRNA on gene expression of chondrocyte-specific markers were analyzed using semi-quantitative RT-PCR. Link N mRNA delivery was accompanied by enhanced expression of *Sox 9* and its transcriptional target type II collagen. Furthermore, in addition to the up-regulation of *aggrecan* and *collagen II* gene expression, the Alcian blue and safranin O staining confirmed the increased production of collagen and proteoglycans. Although primary chondrocytes were used in our experiments, these findings were in accordance with the previous study by Mwale et al. [19], which demonstrated that Link N has the potential to be used in repairing degenerated IVD. Furthermore, discernible down-regulation of the hypertrophic chondrocyte marker collagen X in Link N mRNA transfected cells was also observed compared to untreated cells. Sox 9 as a cartilage anabolic transcription factor could suppress cooperatively with Link N the hypertrophy. Moreover, the up-regulation of *BMP2* and *BMP7* gene expression suggests that the augmented anabolic response in primary chondrocytes upon Link N mRNA transfection might be related to increased BMP synthesis. BMPs including BMP2 and BMP7 have previously been reported to stimulate IVD matrix production in vitro and in vivo [18]. 

In the present study we also investigated the synergy between MSCs and Link N mRNA delivery regarding the stimulation of ECM synthesis. Link N mRNA delivery in SCP1 cells up-regulated chondrocyte-specific marker expression as seen 24 h after transfection. Moreover, Antoniou et al. previously showed that Link N alone cannot drive chondrogenesis in MSCs, but can boost the ongoing process [20]. Besides the potential to enhance the chondrogenic differentiation of MSCs, the negative impact of Link N on osteogenesis and hypertrophy has also been demonstrated in previous studies [19,20], although the exact molecular mechanism of Link N is still not known. In addition, the effect of Link N peptide on MSCs through BMP signaling remains to be investigated. Therefore, in the present study, we evaluated for the first time, the effect of Link N mRNA transfection on Smad signaling in mesenchymal stromal cells. Using Ad5-BRE-Luc reporter (Smad1/5/8 reporter) and Ad5-CAGA9-MLP-Luc reporter constructs (Smad2/3 reporter), we demonstrate that Link N mRNA transfection transiently induces BMP expression but represses BMP signaling. Interestingly, together with TGFβ it boosts Smad 2/3 signaling. The obtained observation corresponds to the previous study by Ehnert et al. [37], showing that TGFβ inhibit BMP responses in cells of the mesenchymal lineage. Besides a tight regulation of BMP–TGFβ-interplay, other pathways involved in the Link N-dependent differentiation of SCP1 cells needs further investigations. Thus, we speculate that the exogenous delivery of Link N mRNA and the presence of unique ECM milieu containing growth factors at the defect site could strengthen the formation of either a disc-like or a cartilage-like matrix. IVT mRNA treatment duration primarily depends on its intended mode of action, concentration, potency, and the circulation half-life of the produced protein. Synthetic mRNA is often degraded in 2–3 days [13], and therefore, a repeated transfection or sustained delivery of nucleic acids is required for prolonged expression of protein [38]. Especially, if a conversion of cell fate for example differentiation of MSCs into chondrocytes [39] or reprogramming of cells to pluripotency [38] is required. Thus, for sustained mRNA delivery and prolonged protein expression, various strategies using nanoparticles [40], hydrogels [41], and biomaterials such as collagen sponges [42] have been investigated. Thereby, the regenerative potential can be further improved.

Due to the lack of a self-healing capability, cartilage and IVD injuries do not completely heal [43]. Most of the current cartilage and IVD treatments fail to fully restore the damaged tissue [43,44,45,46]. Thus, there is a huge need for alternative treatment strategies that can activate the repair and healing process upon and provide mechanical stability. Consequently, in this study, we analyzed a combined approach of synthetic Link N mRNA delivery together with knitted titanium scaffold as a promising strategy for improving and supporting the reparative processes for the treatment of joint tissues. Previously, our group demonstrated that knitted titanium scaffold, which was aimed for IVD nucleus pulposus replacement, supports cell attachment, growth of human primary chondrocytes as well as SCP1 cells in vitro [23]. Importantly, the 3D architecture of the porous knitted titanium scaffold is expected to play a role in maintaining space, which is necessary for tissue integration upon implantation in vivo. In this study, SCP1 cells and human primary chondrocytes seeded on titanium scaffolds were successfully transfected with the synthetic mRNA, which was demonstrated using eGFP mRNA and the subsequent eGFP production in the cells. The consequence of combined use of knitted titanium and Link N mRNA was validated by analyzing the gene expression of chondrocyte-specific markers in chondrocytes and SCP1 cells seeded on knitted titanium scaffold. Link N mRNA transfection of cells seeded on knitted titanium scaffolds resulted in significant up-regulation of chondrocyte-specific genes, confirming the bioactivity of generated Link N mRNA. Cellular behavior in response to exogenous Link N mRNA delivery on titanium scaffold revealed unraveled that the expression levels of chondrocyte-specific genes in 2D and 3D environment was almost alike. However, the 3D structure of the knitted titanium scaffold can further improve the migration, spreading and proliferation of cells and thus influence cell differentiation, organization, and function [47]. Pore size and porosity further positively affect cellular infiltration and diffusion rates of nutrients. Thus, increased Link N production in combination with knitted titanium scaffolds can result in improved long term healing response and the integrity of the scaffold in vivo, thereby reducing aseptic loosening events. To advance this approach to clinical translation, the regeneration potential should be analyzed in animal models upon the delivery of Link N mRNA either unaided or together with a scaffold.

## 4. Materials and Methods

### 4.1. Ethics Statement

All studies involving human participants were performed in accordance with the 1964 Helsinki declaration and its later revisions. Primary human chondrocytes were isolated from femoral condyle tissue explants of patients who received total joint replacement. Tissue was harvested only after medical consultation and patient’s written consent. Corresponding ethical vote (338/2015BO2, dated on 30 June 2015) was approved by the Ethics Commission at the Medical Faculty of Eberhard-Karls University and at the University Hospital Tübingen.

### 4.2. Generation of Modified Synthetic mRNA

Link N and enhanced green fluorescent protein (eGFP) mRNAs were generated as previously reported [48]. Briefly, pcDNA 3.3 plasmid (Aldevron, Fargo, ND, USA) insert encoding eGFP and pEX-K4 plasmid insert encoding Link N (Eurofins Genomics GmbH, Ebersberg, Germany) were amplified using the Hot Star HiFidelity Polymerase Kit (Qiagen, Hilden, Germany), according to manufacturer’s instructions. Therefore, forward (TTGGACCCTCGTACAGAAGCTAA TACG) and reverse (T_120_ CTTCCTACTCAGGCTTTATTCAAAGACCA) primers (Ella Biotech, Martinsried, Germany) were used to amplify the insert. PCR products were then purified using QIAquick PCR Purification Kit (Qiagen, Hilden, Germany) followed by elution in 2 × 10 μL nuclease-free water (Qiagen, Hilden, Germany), and processed for qualitative analysis. Subsequently, in vitro transcription (IVT) was performed to generate mRNA from the DNA product. IVT was conducted using MEGAscript T7 Kit (Ambion, Glasgow, Scotland) and the generated mRNA was modified using 2.5 mM 3’-0-Me-m7G(5’)ppp(5’)G RNA Cap Structure Analog (New England Biolabs, Frankfurt am Main, Germany). Furthermore, 7.5 mM ATP, 1.875 mM GTP (both from MEGAscript T7 Kit), 7.5 mM 5-methylcytidine (5mCTP), 7.5 mM pseudouridine (Ψ) (both from TriLink BioTechnologies, San Diego, CA, USA), and 40 U RiboLock RNase inhibitor (Thermo Scientific, Waltham, MA, USA) were added into the reaction tube. Finally, the product was purified with the RNeasy Kit (Qiagen, Hilden, Germany) and eluted in 2 × 15 μL nuclease-free water. Subsequently, dephosphorylation was performed using the Antarctic Phosphatase Kit (New England Biolabs, Frankfurt am Main, Germany) to remove 5′-triphosphates, and the synthetic mRNA was purified again using the RNeasy Kit in 2 × 40 μL nuclease-free water. The quality of the obtained mRNA was analyzed using 1% agarose gel electrophoresis, and the concentration of the mRNA was measured using a spectrophotometer.

### 4.3. Cy3 Labeling of Link N mRNA

During the IVT of synthetic Link N mRNA, 1.9 mM 5-azido-C3-UTP (Jena Bioscience, Jena, Germany), and 5.6 mM Ψ were used instead of 7.5 mM Ψ and the product was purified using RNeasy MinElute Cleanup Kit. To perform Cu(I)-free azide-(dibenzocyclooctyne [DBCO]) click reaction, 5-fold molar excess of DBCO-sulfo-Cy3 (Jena Bioscience, Jena, Germany) was used to conjugate Cy3 to 5-azido-C3-UTP-modified mRNA in a total amount of 40 µL nuclease-free water for 1 h at 37 °C. Then, the reaction mixture was purified using RNeasy Mini Elute Cleanup Kit and the mRNA concentration was determined using a photometer. The mRNA was stored at −80 °C.

### 4.4. Analysis of Transfection Efficiency Using Cy3 Labeled Link N mRNA

SCP1 cells and primary chondrocytes were transfected with Cy3 labeled synthetic Link N mRNA. After 24 h, cells were washed with DPBS (w/o Ca^2+^/Mg^2+^) and the uptake of Cy3 labeled Link N mRNA was analyzed by using an epifluorescence microscope (Life technologies, Darmstadt, Germany). Furthermore, cells were trypsinized (Trypsin-EDTA; 0.05%/0.02%, Merck, Darmstadt, Germany), centrifuged and resuspended to attain a cell suspension and 20,000 cells were analyzed by FACScan flow cytometer (Sysmex, Norderstedt, Germany). Cells expressing Cy3 were detected using the FL2 channel and fluorescence was measured (Ex/Em  =  554/568 nm). FACS data were displayed in dot plots and respective histograms. For the evaluation, a marker M1 was set, which contained maximum of approximately ≤1.5% of control cells (cells treated with transfection medium without mRNA). FCS5 express flow cytometer software (De novo software, Glendale, CA, USA) was used to determine the percentage of Cy3 positive cells and thereby the transfection efficiency.

### 4.5. Analysis of Cell Viability

SCP1 cells and primary chondrocytes were transfected with synthetic Link N mRNA (1 µg/1 × 10^5^ cells). After 24 h, the cells were detached, loaded into a Via1-Cassete™, which is coated with Acridine Orange and DAPI (ChemoMetec, Kaiserslautern, Germany). Using the NucleoCounter^®^ NC-200™, the cell count and viability were determined. All cells stained with Acridine Orange appeared green and non-viable cells stained with DAPI appeared blue. Cell viability was calculated using the NucleoCounter software.

### 4.6. Cultivation of Cells

Human immortalized bone marrow derived mesenchymal stromal cell line (SCP1 cells) [49] was a kind gift from Prof. Dr. Matthias Schieker. Cell were expanded in culture medium (α-MEM (minimal essential medium; GIBCO, Darmstadt, Germany)), 10% FCS (GIBCO, Darmstadt, Germany), and 1% penicillin/streptomycin (Merck, Darmstadt, Germany) 10,000 units/mL penicillin and 10 mg/mL streptomycin) in a cell incubator at 37 °C and 5% CO_2_ [37].The isolation of primary human chondrocytes was performed as previously described [23]. Briefly, chondrocytes were isolated from donated femoral condyle tissues (donor number N = 23; sex m/f = 10/13, respectively; age = 74.86 ± 8.15 years) (BG Klinik, Tübingen) by collagenase (1500 U/mL, GIBCO, Darmstadt, Germany) digestion, and expanded in culture medium (DMEM/Ham’s F12 (1:1), 10% FCS, 1% penicillin/streptomycin, 50 µM L-ascorbate-2-phosphate (Merck, Darmstadt, Germany) at 37 °C and 5% CO_2_.Different donor samples (N ≥ 3)with passage number 2 were used for each experiment.

### 4.7. Transfection of Cells with Modified Synthetic Link N mRNA in 2D Cell Culture

SCP1 cells and human primary chondrocytes were seeded onto 12-well plate (Corning Costar, Merck, Darmstadt, Germany) with a density of 1 × 10^5^ cells per well and incubated in a cell incubator (Heraeus 6000 Thermo Fischer Scientific, Langenselbold, Germany) overnight at 37 °C and 5% CO_2_. Next day, cells reached a confluency of around 80–90% and the mRNA transfection was performed. Briefly, 1 µg of synthetic mRNA encoding Link N or eGFP was co-incubated with 2 μL Lipofectamine 2000 (Invitrogen, Carlsbad, CA, USA) in 500 μL Opti-MEM I (Invitrogen, Carlsbad, CA, USA) for 20 min at room temperature, allowing the formation of lipoplexes. After the incubation time, cell culture medium was aspirated and cells were washed with 1 mL DPBS (w/o Ca^2+^/Mg^2+^, Merck, Darmstadt, Germany) and then, transfection complexes were added to the cells and incubated initially for 4 h at 37 °C and 5% CO_2_. Subsequently, the transfection solution was replaced with 1 mL of fresh cell culture medium.

### 4.8. Semi-Quantitative RT-PCR

Extracting total RNA was performed using theTriFAST reagent (Peqlab, Erlangen, Germany), according to the manufacturer’s instructions. RNA concentration was measured using spectrophotometer (Omega plate reader, BMG Labtech, Offenburg, Germany) and 1 µg of total RNA was reverse transcribed to generate the complementary DNA (cDNA) with the First Strand cDNA Synthesis Kit (Fermentas, St. Leon-Rot, Germany). PCRs were performed using Biozym Ready Mix (Biozym, Hessisch Oldendorf, Germany). Primer sequences and PCR conditions optimized for each primer set are summarized in Table 1.Representative images from the optimization see Figure S1. *GAPDH* was used as internal control. To obtain PCR results in the linear range 10 ng of cDNA (with the exception of 20 ng for *ACAN*) was used as a template for each set of PCR. PCR products were separated by gel electrophoresis and visualized by ethidium bromide using the pUC19/Msp1 marker (Carl Roth, Karlsruhe, Germany) as a size reference. Densitometric analyses were performed to quantify signal intensities using the ImageJ software (NIH, Bethesda, MD, USA). 

### 4.9. Histochemical Analysis of ECM Production

Glycosaminoglycans (GAGs) and collagen are the primary matrix constituents of articular cartilage and IVDs and confer tensile and compressive integrity to these joints [50]. Thus, to assess the influence of the exogenous delivery of synthetic Link N mRNA on GAGs and collagen synthesis, human primary chondrocytes were transfected with (1 µg/1 × 10^5^ cells) Link N mRNA. After 7 days of post-transfection, alcian blue and safranin O staining were performed. Briefly, after the removal of cell culture medium, cells were fixed in 4% paraformaldehyde for 15 min and then washed twice with DPBS. Subsequently, 1% Alcian blue (in 3% acetic acid) or 0.1% safranin O (in distilled water) stain (Carl Roth, Karlsruhe, Germany) were added and incubated at room temperature for 30 min. Afterwards, dye solution was removed and cells were washed with distilled water. The stained cells were imaged using EVOS_fl_ microscope (Life technologies, Darmstadt, Germany).

### 4.10. Dot Blot Assay for Quantifying Link N Protein Post mRNA Transfection

Briefly, supernatants of Lipofectamine 2000 alone, eGFP mRNA, or Link N mRNA-transfected SCP1 cells and chondrocytes were collected after 24 h. Protein concentrations were determined with the Lowry assay and 10 μg of cell supernatant was pipette onto the nitrocellulose membrane and allowed to air dry. Membranes were then blocked with 5% BSA in TBST for 1 h, followed by 3 washing steps (10 min each). Then, the membranes were incubated overnight with HAPLN1 (TA325115-Origene, Herford, Germany) primary antibody (dilution 1:1000) at 4 °C, and the next day membranes were washed 3 times (10 min each). Afterwards, the membrane was treated with HRP-conjugated secondary anti-rabbit IgG (Santa cruz, dilution 1:10,000) for 2 h. Membranes were developed upon incubating them with ECL substrate solution for a minute. Chemiluminescent signals were quantified using image J analysis software 

### 4.11. Transient Cell Infections and Gene Reporter Assay

Smad 1/5/8 reporter adenovirus particles (Ad5-BRE-Luc) and/or Smad2/3 reporter adenovirusparticles (Ad5-CAGA9-MLP-Luc) provided by Prof. P. ten Dijke were used to infect cells as described before [37]. Activation of phosphorylated Smad1/5/8 and/or Smad2/3 allows luciferase expression in the cell cytoplasm. Cell lysates were then prepared and luciferase activity was measured according to the manufacturer’s instructions, using the Steady-Glo Luciferase Assay System (Promega, Madison, WI, USA). Measurements were normalized to total protein content. Ad5-GFP (green fluorescent protein) was used as a positive control where, infection efficiency was shown to be >90% by fluorescent microscopy (24 h).

### 4.12. Preparation of Knitted Titanium Scaffolds and Cell Seeding

Briefly, medical grade titanium alloy (Ti6Al4V) wires with a diameter of 0.25 mm were knitted to produce a mesh like cuboidal disc structure, with a dimension of 14 × 11 × 5 mm, a volumetric porosity of 67.67 ± 0.824%, and a density of 0.75 mg/cm^3^.These knitted titanium scaffolds (Buck GmbH & CO., Bondorf, Germany), which showed in our previous study [22,23] a high cytocompatibility, were used for the 3D cultivation of chondrocytes and SCP1 cells. Therefore, 2.0 × 10^5^ cells were seeded per 50 µL cell culture medium per scaffold and incubated initially for 3 h at 37 °C to allow cell attachment. Afterwards, culture medium was added and seeded scaffolds were further incubated at 37 °C and 5% CO_2_. This seeding protocol was identified as the optimal way to facilitate cell attachment in our previous study [22,23].

### 4.13. Transfection of Cells Seeded on Knitted Titanium Scaffold

The transfection of cells with synthetic Link N mRNA was performed 24 h after the seeding of the scaffolds with cells. Therefore, 1 µg of mRNA was co-incubated with 2 µL of Lipofectamine 2000 in 50 µL Opti-MEM for 20 min at room temperature, allowing the formation of lipoplexes. After the incubation time, cell culture medium was aspirated and cells seeded on the scaffold were washed with 1 mL DPBS (w/o Ca^2+^/Mg^2+^, Sigma, Munich, Germany). Transfection complexes were then added and incubated initially for 4 h at 37 °C and 5% CO_2_. Subsequently, 750 µL of fresh cell culture medium was added and scaffolds were incubated at 37 °C and 5% CO_2_. eGFP mRNA was used as a positive control to confirm the transfection. Functional potency of synthetic Link N mRNA was examined by evaluating chondrocyte-specific gene expression, 24 h post transfection.

### 4.14. Statistical Analysis

Analysis was performed using GraphPad Prism 5.0 software (El Camino Real, CA, USA) and PAST.exe (http://folk.uio.no/ohammer/past/index). All experiments were conducted at least three times, independently (N ≥ 3) and results are represented as mean ± standard error of mean (SEM). Statistical significance was compared using one-way ANOVA with Bonferroni’s multiple comparison test (*** *p* < 0.001, ** *p* < 0.01, * *p* < 0.05).

## 5. Conclusions

In this study, the synthetic modified Link N mRNA was successfully generated, and its delivery into chondrocytes and SCP1 cells resulted in an anabolic response augmentation. Our results suggest that Link N has a regulatory function in the BMP–TGFβ interplay is required for chondrogenic differentiation. In combination with a knitted titanium scaffold, Link N mRNA delivery also led to increased expression of chondrocyte-specific markers. Therefore, we suppose that the application of Link N mRNA therapeutics to the field of musculoskeletal tissue engineering either in combination with or without a scaffold offers a promising forthcoming direction for the repair of articular cartilage and IVD. The present study provides a first step toward mRNA-based approach to improve musculoskeletal regeneration.

## Figures and Tables

**Table 1:** List of used primers, product lengths, annealing temperatures.

Gene Name	Accession Number	Forward Primer (5′-3′)	Reverse Primer (3′-5′)	Amplicon (bp)	T_m_ (°C)	Cycle No.
***GAPDH***	NM_002046	GTCAGTGGTGGACCTGACCT	AGGGGTCTACATGGCAACTG	420	56	30
***SOX9***	NM_000346	GAAGGACCACCCGGATTACA	GCCTTGAAGATGGCGTTGG	120	60	35
***ACAN***	NM_001135	CTTGGACTTGGGCAAACTGC	CACTAAAGTCAGGCAGGCCA	143	60	35
***COL X***	NM_000493	AAACCTGGACAACAGGGACC	CGACCAGGAGCACCATATCC	125	60	35
***COL II***	NM_001844	TGGATGCCACACTCAAGTCC	GCTGCTCCACCAGTTCTTCT	254	60	35
***BMP2***	NM_001200	CCCCCTACATGCTAGACCTGT	CACTCGTTTCTGGTAGTTCTTCC	150	60	35
***BMP7***	NM_001719	TAGCCATTTCCTCACCGACG	AGATCCGATTCCCTGCCCAA	255	60	35

## Supplementary Materials

Supplementary materials can be found at https://www.mdpi.com/1422-0067/20/7/1716/s1.
