# Supplementary material for: Exogenous Delivery of Link N mRNA into Chondrocytes and MSCs—The Potential Role in Increasing Anabolic Response
Source: Int J Mol Sci. 2019 Apr 6;20(7):1716. doi: 10.3390/ijms20071716 (PMC6479841; doi:10.3390/ijms20071716)
Supplement: Supplementary file 1 [file ijms-20-01716-s001.pdf]

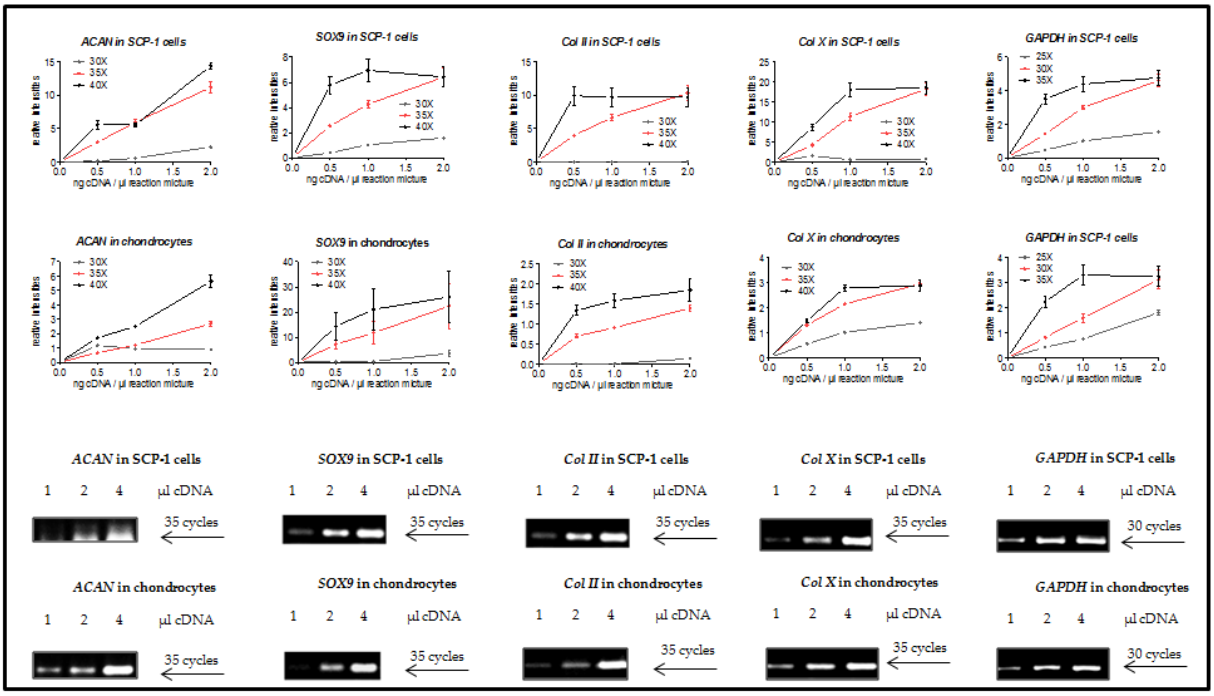

Figure S1. Primer optimization for semi-quantitative RT-PCR of aggrecan (*ACAN*), *Sox 9*, type II and X collagen. *GAPDH* was used as house-keeping gene. PCRs were performed with cDNA (concentration: 10 ng/µl) from SCP-1 cells and primary chondrocytes in the range of 1, 2 and 4 µl. PCR was performed with 30, 35 and 40 cycles each.
